# Variability in quantifying the Hill-Sachs lesion: A scoping review[Author-notes fn1-17585732221123313]

**DOI:** 10.1177/17585732221123313

**Published:** 2022-09-13

**Authors:** Shahrukh Khan, Ajaykumar Shanmugaraj, Haseeb Faisal, Carlos Prada, Sohaib Munir, Timothy Leroux, Moin Khan

**Affiliations:** 1 Faculty of Health Sciences, 3710 McMaster University, Hamilton, ON, Canada; 2 Faculty of Medicine, University of British Columbia, Vancouver, BC, Canada; 3 Division of Orthopaedic Surgery, 3710 McMaster University, Ontario, Canada; 4 Department of Radiology, 3710 McMaster University, Ontario, Canada; 5 Division of Orthopaedic Surgery, 3710 University of Toronto, Toronto, Ontario, Canada; 6 Department of Health Research Methods, Evidence, and Impact, 3710 McMaster University, Hamilton, ON, Canada

**Keywords:** Hill-Sachs lesion, shoulder, glenohumeral, radiography, modalities, measurement

## Abstract

**Background:**

Currently, is there no consensus on a widely accepted measurement technique for calculating the Hill-Sachs lesion (HSL). The purpose of this review is to provide an overview of the techniques and imaging modalities to assess the HSL pre-operatively.

**Methods:**

Four online databases (PubMed, Embase, MEDLINE, and COCHRANE) were searched for literature on the various modalities and measurement techniques used for quantifying HSLs, from data inception to 20 November 2021. The Methodological Index for Non-Randomized Studies tool was used to assess study quality.

**Results:**

Forty-five studies encompassing 3413 patients were included in this review. MRA and MRI showed the highest sensitivity, specificity, and accuracy values. Intrarater and interrater agreement was shown to be the highest amongst MRA. The most common reference tests for measuring the HSL were arthroscopy, radiography, arthro-CT, and surgical techniques.

**Conclusion:**

MRA and MRI are reliable imaging modalities with good test diagnostic properties for assessment of HSLs. There is a wide variety of measurement techniques and imaging modalities for HSL assessment, however a lack of comparative studies exists. Thus, it is not possible to comment on the superiority of one technique over another. Future studies comparing imaging modalities and measurement techniques are needed that incorporate a cost-benefit analysis.

## Introduction

A Hill-Sachs lesion (HSL) is categorized as a bony defect of the posterosuperolateral humeral head, often caused by prior episodes of anteroinferior glenohumeral dislocation.^1,2^ Recurrent instability at the glenohumeral joint is often observed after a HSL due to anterior glenoid impact by the posterolateral aspect of the humeral head resulting in subsequent pain and difficulty moving the shoulder joint.^1,3^ The clinical prevalence of HSLs range from 70% to 90% after an anterior shoulder dislocation and may approach up to 100% incidence rate in patients with recurrent anterior shoulder instability.^4–7^

Measurement of the HSL has been an area of interest for clinicians as quantification of bone loss is crucial in treatment decisions for patients with shoulder instability. Currently, various modalities can be used to measure a HSL such as computed tomography (CT) scans, magnetic resonance imaging (MRI), 3D CT and 3D MRI, magnetic resonance arthrography (MRA), among many others.^8,9^ In addition to the variety in imaging modalities, various measurement methods are currently available to evaluate and quantify the HSL such as length, width, and depth measurements.^8–10^ Measurement methods such as the renowned “on-track” and “off-track” concept utilizes the glenoid track, which consists of the contact area between the humeral head and glenoid during shoulder abduction and external rotation. This method determines whether the HSL engages the anterior glenoid rim resulting in shoulder dislocation, where it is termed “off-track,” or does not engage, known as “on-track.”^11^

Currently, there is extensive literature reporting imaging agreement of other specific measurements such as glenoid bone loss. Most notably, Walter et al. determined the most accurate imaging techniques in measuring glenoid bone loss in anterior glenohumeral instability.^12^ Despite the various modalities and methods available to measure the HSL, challenges still exist upon evaluation related to its 3D aspect of the humeral sphere and conflicting visibility during imaging, with each method having its own pros and cons along with varying degrees of reliability.^13–16^ This acts as a major barrier as minor changes in measurements in the context of HSLs when dealing with bipolar bone lesions in shoulder instability can have significant implications in patient's surgical treatment. Henceforth, the purpose of this review is to provide an overview of the imaging modalities and techniques to measure the HSL and to assess their diagnostic properties. It was hypothesized that 3D-computed tomography (3D-CT) and/or 3D MRI would be the most prevalent and reliable imaging modality to quantify the HSL.

## Methods

### Search strategy

The search terms included “shoulder,” “Hill-Sachs,” “bone loss,” and similar phrases (Appendix Table 1). PUBMED, EMBASE, MEDLINE, and COCHRANE databases were searched for literature on the reliability of imaging modalities and measurement techniques for quantifying the HSL from database inception to 20 November 2021. The search terms were then entered into Google Scholar to ensure that articles were not missed. Inclusion criteria were (1) HSL; (2) quantification by imaging modalities; (3) present a method for measuring HSLs; (4) human studies; and (5) English language. The exclusion criteria were: (1) measurement of other major shoulder pathologies (e.g. glenohumeral, Bankart lesions) without mention of an HSL; (2) review articles; (3) non-imaging studies; (4) cadaver studies; (5) case reports and editorials.

### Study screening

Systematic screening was in compliance with Preferred Reporting Items for Systematic Reviews and Meta-analyses (PRISMA) and Revised Assessment of Multiple Systematic Reviews (R-AMSTAR) guidelines.^17,18^ Two reviewers (S.K., H.F.) independently screened the titles and abstract, and full-texts in duplicate. Discrepancies were discussed and resolved with input of a third reviewer (A.S). The references of included studies were also screened using the same systematic approach to capture any additional relevant articles.

### Data abstraction

Data were extracted independently by two reviewers (S.K., H.F.) who abstracted relevant data from included articles, recording onto a spreadsheet in Microsoft Excel (Version 2016; Microsoft, Redmond, Washington) designed *a priori*. Authors were contacted for clarification if data was unclear or not reported. Extracted data included, but were not limited to, year and journal of publication, sample size, study design, level of evidence, and patient demographics (e.g. gender, age, etc.). Information regarding modality and measurement techniques, reliability, and tests diagnostic properties when present were documented.

### Statistical analysis

Due to high statistical and methodological heterogeneity, a meta-analysis could not be performed, and the results are summarized descriptively. Descriptive statistics such as mean, range, and measures of variance (e.g. standard deviations, 95% confidence intervals [CI]) are presented where applicable. The intraclass correlation coefficient (ICC) was used to evaluate inter-reviewer agreement for assessing study quality. A kappa (κ) statistic was used to evaluate inter-reviewer agreement at all screening stages. Agreement was categorized a priori as follows: ICC/κ of 0.81 to 0.99 was considered as almost perfect agreement; ICC/κ of 0.61 to 0.80 was substantial agreement; ICC/κ of 0.41 to 0.60 was moderate agreement; 0.21 to 0.40 fair agreement and a ICC/κ value of 0.20 or less was considered slight agreement.^19^ Statistics were performed using Microsoft Excel (Version 2016; Microsoft, Redmond, Washington).

### Quality assessment

The methodological quality of non-randomized studies was evaluated using the methodological index for nonrandomized studies (MINORS).^14^ A score of 0, 1, or 2 is given for each of the 12 items on the MINORS checklist with a maximum score of 16 for non-comparative studies and 24 for comparative studies. Methodological quality was categorized a priori as follows: a score of 0–8 or 0–12 was considered poor quality, 9–12 or 13–18 was considered fair quality, and 13–16 or 19–24 was considered excellent quality, for non-comparative and comparative studies, respectively.

## Results

### Study characteristics

**Figure 1.**
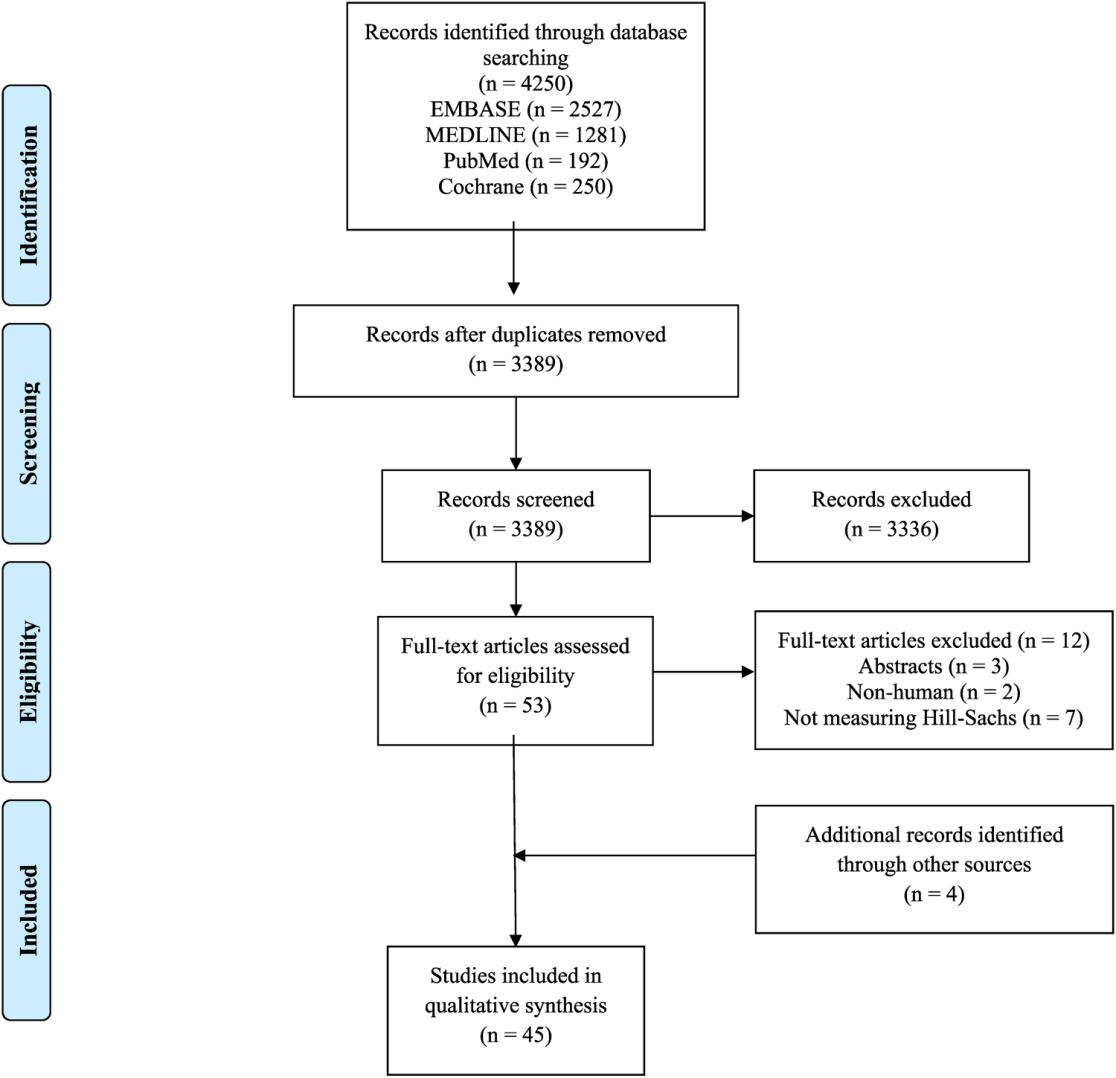
PRISMA flow diagram.

The initial search on the topic yielded a total of 4250 articles. After removing 861 duplicates, a systematic screening process yielded 45 articles that met inclusion criteria (Figure 1). One study was found upon reviewing references of included studies. Of the included studies, there were 19 retrospective cohort (42%), 18 prospective cohort (40%), and seven other studies (16%). One of the included studies was a conference abstract (2%) (Table 1).

**Table 1.** Study characteristics and methodological quality.

Study	Study design (level of evidence)	Sample size	Mean age	Index tests	Reference test	MINORS score*
Stefaniak J. (2020)	Retrospective cohort (III)	100	35.5 ± 15.5	CT and 3D CT	NR	22/24
Beason A.M. (2019)	Retrospective cohort (III)	40	NR	Plain radiographs and MRI	NR	17.5/24
Chalmers P.N. (2020)	Retrospective cohort study (III)	53	NR	MRI vs CT	NR	22/24
Stillwater L. (2017)	Retrospective cohort (III)	12	29	3D-MR vs 3D-CT	NR	22/24
Assuncao J.H. (2017)	Retrospective case series (IV)	50	25.3 ± 4.7	CT and CTA	NR	13/16
Schneider A.K. (2017)	Biomechanical study	71	25.5	3D-CT	NR	12.5/16
Gyftopoulos S. (2015) MRI evaluation of bipolar bone loss using the on-track off-track method: A feasibility study.	Prospective cohort (II)	75 (76 shoulders)	30	MRI	Arthroscopy	16/16
Kinsella S.D. (2015)	Prospective cohort (IV)	30	16.3 and 16.4	MRA	NR	13.5/16
Gyftopoulos S. (2015) MRI evaluation of bipolar bone loss: Can it be used to predict failure of arthroscopic shoulder stabilization?	Retrospective cohort (II)	39 (40 shoulders)	31.7	MRI	NR	11/16
Horst K. (2014)	Retrospective cohort (III)	105	male: 32.6 ± 14.0 (16–74) female: 48.2 ± 20.3 (17–80)	MRI	NR	13.5/16
Bernhardson A. (2014)	Abstract	217	NR	3D-CT	NR	10/16
Ozaki R. (2014)	Retrospective cohort (II)	135	20.2	3D-CT	Arthroscopy	14/16
Pavic R. (2013)	Retrospective cohort (III)	200	39	US MRI MRA	Arthroscopy	17/24
Mahmoud M.K. (2013)	Prospective cohort (II)	31	NR	MRA	Arthroscopy	19/24
Auffarth A. (2013)	Retrospective cohort (III)	20	NR	CT	Radiographs	15/16
Chauvin N.A. (2013)	Retrospective cohort (III)	66	Boys: 15.4 Girls: 15.6	MRA	arthroscopy	15/16
Hardy P. (2012)	Prospective cohort (II)	71	29	CTA and X-ray	Arthroscopy	21/24
Cho S.H. (2011)	Prospective cohort (II)	104	23.7	3D-CT	Arthroscopy	14/16
Hayes M.L. (2010)	Retrospective cohort (III)	87	27	MRI	Arthroscopy	14/16
Oh J.H. (2010)	Prospective cohort (II)	148	46.6	CTA and MRA	Arthroscopy	20.5/24
Saito H. (2009)	Retrospective case control (IV)	34 (35 shoulders)	28	CT	NR	12/16
van Grinsven S. (2007)	Retrospective cohort (III)	61	29	MRA	Arthroscopy or open surgery	16/16
Matheson G.O. (2004)	Prospective cohort (II)	16	21.6	MRI	Arthroscopy	15.5/16
Willemsen U.F. (1998)	Prospective cohort (II)	44	30	MRA	Arthroscopy or open surgery	11.5/16
Wintzell G. (1998)	Prospective cohort (II)	16	24 + 4	MRA	Diagnostic arthroscopy or open stabilizing operation	12/16
Pancione L. (1997)	Prospective cohort (II)	60	32	US	Arthro-CT	12/16
Workman T.L. (1992)	Retrospective case series (IV)	76	28	MRI	Radiographs/arthroscopy (substituted both in group with index as well)	13/16
Funakoshi T (2019)	Retrospective case series (IV)	16	32.4 ± 2.5	3D-CT	Arthroscopy	15.5/16
Shijith KP (2019)	Retrospective cohort (III)	44	25.95 ± 4.2	CT	NR	15/16
Breighner RE (2018)	Prospective cohort (II)	34	40 ± 22	CT and MRI	NR	21/24
Probyn LJ (2007)	Retrospective cohort (III)	39	28	MRA	Arthroscopic and open procedures	15/16
Farin PU (1996)	Prospective cohort (II)	86	36	US and CTA	Arthroscopy	17.5/24
Kirkley, A (2003)	Prospective cohort (II)	16	21.6	MRI	Arthroscopy	13.5/16
Charousset et al. (2010)	Retrospective case series (IV)	57	NR	CTA	AP radiograph	10/16
Fogerty et al (2011)	Retrospective cohort (III)	50	34.9	CTA	NR	13/16
O’Brien et al (2012)	Retrospective cohort (III)	165	NR	MRA	NR	15.5/16
Saqib et al (2017)	Retrospective cohort (III)	194	29.9	MRA	Arthroscopy	12.5/16
Simão et al (2012)	Prospective cohort (II)	56	26.3	US and MRA	Arthroscopy	21.5/24
Suder et al. (1995)	Prospective cohort (II)	25	27	MRI	Arthroscopy	13.5/16
Tirman et al. (1993)	Prospective cohort (II)	65	NR	MRA	Arthroscopy or open surgical therapy	9/16
Cicak et al (1998)	Prospective cohort (II)	61	NR	US and radiography	Surgery	14/16
Shaha et al. (2016)	Prospective cohort (II)	57	25.5	MRI	Arthroscopy/surgery	16/16
Sgroi et al. (2021)	Retrospective cohort (III)	50	26 ± 11.8	CT and MRI	Arthroscopy	
Riebe et al. (2021)	Retrospective cohort (III)	181	31.4	Radiographs	MRI	
Yu et al. (2020)	Retrospective cohort (III)	256	31.2	Radiographs	MRI	

NR: not reported.

### Study quality

There was substantial agreement between the reviewers for title and abstract screening (κ  =  0.759; 95% CI 0.461–0.877), and almost perfect agreement for full-text screening (κ  =  0.838; 95% CI 0.662–1.000). The majority of the studies (44%; *n*  =  19) were level II evidence, whereas 15 studies (42%; *n*  =  18) were level III evidence, and six studies (14%; *n*  =  6) were level IV evidence. The mean MINORS's scores across comparative and non-comparative studies are 20.1 and 9.9, respectively, indicating excellent and fair quality of evidence, respectively. Furthermore, there was excellent agreement between the raters in their classification of these studies (ICC  =  0.99; 95% CI 0.99–0.99) (Table 1).

### Distribution of modalities used and reference tests

Among the 45 studies, the distribution of modalities was MRA (23%), MRI (23%), 3D-CT (13%), CT (11%), computerized arthrotomography (CTA) (9%), ultrasound (US) (9%), radiography (5%), 3D-magnetic resonance (3D-MRI) (2%) as an index test. Twenty-seven out of the 42 studies (64%) reported using a reference test. The distribution of reference tests was arthroscopy (*n*  =  23; 63.8%), surgical techniques (*n*  =  7; 19.4%), radiographs (*n*  =  3; 8.3%), MRI (*n*  =  2; 5.5%), and arthro-CT (*n*  =  1; 2.8%) (Table 1).

### Patient characteristics

A total of 3413 patients and 3431 shoulders were included in this review.^8–10,12,13,20–59^ Of the included patients, 74% (1974 out of 2672) were male; nine studies (21%) did not report on gender distribution. The mean age was 28.8  ±  6.3 years, calculated from 37 studies (82%); eight studies (18%) did not report on age.^13,21,24,25,45,49,50,55^

### Measurement techniques

#### Computed tomography (CT)

Reported measurement techniques varied amongst the studies as well as modalities.

For CT, humeral residual articular arc and percentage of articular arc loss, HSL width and depth, percentage of anterior glenoid defect, bare area, on-track and off-track, Franceschi grading, Calandra classification, Richards grading, Hall grading, Rowe grading, Flatow percentage, linear-based and area-based methods were used.^10,31,39,52^ The reported sensitivity when confirmed by CT was between 20% and 65%, and the specificity was between 41.7% and 87%.^25,39^ Accuracy, positive predictive value (PPV), and negative predictive value (NPV) were not reported amongst the CT studies. Intrarater agreement was only reported in one study, where there was 33% agreement for on-track measurements.^56^ Interrater agreement ranged between 41% and 76% (Table 2).^10,25,40,52,56^ Radiographs were the only reference tests that were reported.^25^ For 3D CT, circle area, height, and length of humeral head, HSL length and depth, anatomical neck width, on-track and off-track, and clock-face methods were reported.^8,9,21,22,28,38,57^ Only one study included data on sensitivity, specificity, PPV, and NPV values using the Calandra method.^22^ The values reported were 76.3%, 100%, 100%, and 46.2%, respectively.^22^ One study reported ICC values ranging from 0.92 and 0.99 for intrarater agreement.^28^ Likewise, this study also reported ICC values for interrater agreement, ranging from 0.77 and 0.99 (Table 2). Arthroscopy was the only reference test reported.^9,21,22,28^

**Table 2.** Detecting the presence of Hill-Sachs lesions with computed tomography (CT).

Article	Modality	Reference test	Quantification method	Sensitivity, %	Specificity, %	Accuracy, %	PPV, %	NPV %	Intrarater agreement	Interrater agreement
Assuncao J.H. (2017)	CT or CTA	NR	Humeral residual articular arc and percentage of articular arc loss; Hill-Sachs lesion (HSL) width and depth, percentage of anterior glenoid defect	NR	NR	NR	NR	NR	NR	Range of 0.410–0.731
Auffarth A. (2013)	CT	Radiographs	NR	20.0	87.0	NR	NR	NR	NR	CT = 72% Radiograph = 83%
Saito H. (2009)	CT	NR	Bare area to find size, location, depth, etc.	NR	NR	NR	NR	NR	NR	NR
Shijith KP (2019)	CT	NR	On track off track % bone loss was >9.60%	65.0	41.7	NR	NR	NR	NR	NR
Stefaniak J (2020)	CT and 3D CT	NR	Circle area of humeral head, HSL length, HSL depth, Humeral head length, humeral head height, anatomical neck width (refer to Table 1 for description)	NR	NR	NR	NR	NR	NR	NR
Schneider A.K. (2017)	3D CT	NR	On-track off-track	NR	NR	NR	NR	NR	NR	On-track or off-track = 72% Treatment classification = 65% Coefficient of variability = 19.2%
Bernhardson A. (2014)	3D CT	NR	Measured using 3D models, location of lesion was measured by using anterior margins of the lesion in relation to the biceps groove to give a clock face location. Height, depth and length was record in its greatest dimension.	NR	NR	NR	NR	NR	NR	NR
Ozaki R. (2014)	3D CT	Arthroscopy	Calandra Method: Measured the length and width on 3D CT scans reconstructed with elimination of the scapula, while the depth was measured on axial images obtained perpendicular to the longitudinal axis of the humeral shaft. Hill–Sachs lesions confirmed at arthroscopy were re-examined by the observer who performed the first evaluation.	76.3	100.0	NR	100.0	46.2	NR	NR
Cho S.H. (2011)	3D CT	Arthroscopy	Virtual circle draw on axial CT image on the articular surface of the humeral head, width defined as distance between both ends of the Hill-Sachs lesion where the bone defect was located on the circle	NR	NR	NR	NR	NR	ICC = 0.916–0.999	ICC = 0.772–0.996
Funakoshi T (2019)	3D CT	Arthroscopy	On-track off-track	NR	NR	NR	NR	NR	NR	NR

NR: not reported.

#### Computed arthrotomography (CTA)

For CTA, HSL depth, P/R (notch defect/radius) index calculation, and normal base area measurement were reported.^13,27,42^ When confirmed by CTA, the reported sensitivity ranged between 20% and 93%, whereas the specificity ranged between 90% and 95%. Accuracy, PPV, and NPV were reported in only one study, where it was 90%, 67%, and 98%, respectively.^30^ Intrarater and interrater agreements were only reported in one study, where it was κ  =  0.71, and κ  =  0.30, respectively (Table 3).^13^ The distribution of reference tests used was arthroscopy and AP radiographs.^13,27,30^

**Table 3.** Detecting the presence of Hill-Sachs lesions with computed arthrotomography (CTA).

Article	Modality	Reference test	Quantification method	Sensitivity, %	Specificity, %	Accuracy, %	PPV, %	NPV %	Intrarater agreement	Interrater agreement
Hardy P. (2012)	CTA and X-ray	arthroscopy	Measured depth of lesion and the radius of humeral head on the AP X-ray in 45degree internal rotation	84.0	NR	NR	NR	NR	NR	NR
Charousset et al (2010)	CTA	AP radiograph	P/R index calculation to evaluate the depth of the lesion	NR	NR	NR	NR	NR	K = 0.705	K = 0.30
Fogerty et al (2011)	CTA	NR	NR	NR	NR	NR	NR	NR	NR	K = 0.37

NR: not reported.

#### Magnetic resonance imaging (MRI)

For MRI, on-track and off-track, linear-based and area-based methods, as well as a modified Cetik method were reported.^12,20,40,51,56,58^ When confirmed by MRIs, the sensitivity values ranged between 16.7% and 96.3%, whereas the specificity values ranged from 67% to 100%.^12,29,37,48,58^ Accuracy of detecting the presence of HSLs with MRI ranged from 67% to 88%.^37,48,58^ PPV ranged from 14% to 65%, whereas NPV ranged from 85% to 91%.^12,58^ Intrarater agreement ranged from 41% to 86%.^58^ Interrater agreement ranged from ICC  =  0.33 to 1.00 (Table 4).^43^ The distribution of reference tests was arthroscopy, radiographs, and surgical techniques.^23,29,33,37,43,48,51,58^

**Table 4.** Detecting the presence of Hill-Sachs lesions with magnetic resonance imaging (MRI).

Article	Modality	Reference test	Quantification method	Sensitivity, %	Specificity, %	Accuracy, %	PPV, %	NPV, %	Intrarater agreement	Interrater agreement
Gyftopoulos S. (2015) MRI evaluation of bipolar bone loss using the on-track off-track method: A feasibility study.	MRI	Arthroscopy	On-track and off-track	72.2	87.9	84.2	65.0	91.1	K = 0.86	K = 0.79
Gyftopoulos S. (2015) MRI evaluation of bipolar bone loss: Can it be used to predict failure of arthroscopic shoulder stabilization?	MRI	NR	On-track and off-track	16.7	82.4	NR	14.3	84.9	NR	NR
Horst K. (2014)	MRI	NR	Modified Cetik method and classified according to Calandra	NR	NR	NR	NR	NR	NR	NR
Hayes M.L. (2010)	MRI	Arthroscopy	NR	96.3	90.6	NR	NR	NR	NR	NR
Matheson G.O. (2004)	MRI	Arthroscopy	NR	NR	NR	NR	NR	NR	NR	NR
Workman T.L. (1992)	MRI	Radiographs/arthroscopy	NR	***Arthroscopy reference:*** MRI = 91%, Radiography = 65% ***Radiography reference:*** MRI = 76% Arthroscopy = 43% ***Final Diagnosis:*** MRI = 97% Radiography = 84% Arthroscopy = 73%	***Arthroscopy reference:*** MRI = 72% Radiography = 67% ***Radiography reference:*** MRI = 80% Arthroscopy = 83% ***Final Diagnosis:*** MRI = 91% Radiography = 83% Arthroscopy = 97%	***Arthroscopy reference:*** MRI = 78% Radiography = 67% ***Radiography reference:*** MRI = 78% Arthroscopy = 67% ***Final Diagnosis:*** MRI = 94% Radiography = 83% Arthroscopy = 83%	NR	NR	NR	NR
Kirkley, A (2003)	MRI	Arthroscopy	NR	NR	NR	NR	NR	NR	NR	K = 1.0
Suder et al. (1995)	MRI	Arthroscopy	NR	80.0	100.0	88.0	NR	NR	NR	NR
Shaha et al. (2016)	MRI	Arthroscopy/surgery	On-track off-track	NR	NR	NR	NR	NR	NR	NR

NR: not reported.

#### Magnetic resonance arthrography

For MRA, a method described by O’Brien et al. where measurements of the humeral circumference, as well as the depth of the HSL were used in analysis of the lesion as the most accurate reflection of Hill-Sachs volume.^45^ Sensitivity values for detecting the presence of HSLs with MRA ranged from 69% to 100%.^24,26,30,32,34,41,46,47,49^ Specificity values ranged from 0% to 100%.^24,26,30,32,34,41,46,47,49^ Accuracy varied from 81% to 100%, PPV from 45% to 100%, and NPV from 88% to 100%.^24,30,32,34,41,46,47^ Intrarater agreement was only reported in one study to be ICC  =  1.00, and interrater agreement to be ICC  =  0.97 (Table 5).^45^ The distribution of reference tests was arthroscopy and surgical techniques.^23,24,26,30,32,34,35,41,46,47,49^

**Table 5.** Detecting the presence of Hill-Sachs lesions with magnetic resonance arthrography (MRA).

Article	Modality	Reference test	Quantification method	Sensitivity, %	Specificity, %	Accuracy, %	PPV, %	NPV, %	Intrarater agreement	Interrater agreement
Kinsella S.D. (2015)	MRA	NR	NR	NR	NR	NR	NR	NR	NR	NR
Mahmoud M.K. (2013)	Magnetic Resonance Arthrography (MRA) Multidetector Tomography Arthrography (MDCTA)	Arthroscopy	NR	100.0	100.0	100.0	100.0	100.0	NR	NR
Chauvin N.A. (2013)	MRA	Arthroscopy	NR	100.0	94.0	NR	97.0	NR	NR	NR
van Grinsven S. (2007)	MRA	Arthroscopy or open surgery	NR	70.0	72.0	NR	45.0	88.0	NR	NR
Willemsen U.F. (1998)	MRA	Arthroscopy or open surgery	NR	95.0	50.0	81.0	NR	NR	NR	NR
Wintzell G. (1998)	MRA	Diagnostic arthroscopy or open stabilizing operation	NR	NR	NR	NR	NR	NR	NR	NR
Probyn LJ (2007)	MRA	Arthroscopic and open procedures	NR	100	0	93.0	93.0	NR	NR	NR
O’Brien et al (2012)	MRA	NR	Brooks Hill et al. Measurements of each of the Hill-Sachs lesions were undertaken on the second most superior transverse image, measuring the humeral circumference at this level, as well as the depth of the Hill-Sachs lesion This depth measurement was used in the analysis of Hill Sachs lesions as it was described [Brooks Hill et al., presented at the Canadian Academy for Sport Medicine, Annual Meeting 2009] as the most accurate reflection of Hill Sachs volume.	NR	NR	NR	NR	NR	ICC = 1	ICC = 0.964
Saqib et al (2017)	MRA	Arthroscopy	NR	91.0	91.0	NR	68.0	98.0	NR	NR
Tirman et al. (1993)	MRA	Arthroscopy or open surgical therapy	NR	69.0	87.0	NR	NR	NR	NR	NR

NR: not reported.

#### Ultrasound

For US, only one method was reported as a calculation of the Hill-Sachs volume using V  =  4/3 π 1/2a 1/2bc, where a, b, and c represent the width, length, and depth of the lesion, respectively.^50^

Only one study reported values for sensitivity (95.6%), specificity (92.8%), and accuracy (95.0%).^36^ There were no reported values for both intrarater and interrater agreement (Table 6). The distribution of reference tests was arthroscopy, arthro-CT, and surgical techniques.^23,36,42,47,50^

**Table 6.** Detecting the presence of Hill-Sachs lesions with ultrasound (US).

Article	Modality	Reference test	Quantification method	Sensitivity, %	Specificity, %	Accuracy, %	PPV, %	NPV, %	Intrarater agreement	Interrater agreement
Pancione L. (1997)	US	Arthro-CT	NR	95.6	92.8	95.0	NR	NR	NR	NR
Cicak et al (1998)	US/radiograph	Surgery	V = 4/3 π 1/2a 1/2bc, where a, b, and c represent the width, length, and depth of the lesion, respectively	NR	NR	NR	NR	NR	NR	NR

NR: not reported.

### Clinically relevant bone loss

This scoping review was able to identify four studies (10%) that reported glenoid bone loss percentages ranging from 8.9% to 23.5%.^9,10,56,58^ Threshold values varied among the modalities used in the studies. Hardy et al. assessed a threshold value for making a precise risk factor for failure after an arthroscopic stabilization procedure.^27^ A ratio between depth of the Hill-Sachs lesion (D) and the humeral head radius (R) from conventional radiograph was analyzed, it was found that when the D/R ratio threshold was more than 15%, the failure rate was 56% contrary to only 16% failure when the D/R ratio was less than 15%. Stefaniak et al. determined that for CT measurements, good or moderate ICC values were observed and “reasonable” or above threshold values of 30% of minimal detectable change (MDC 95%).^8^ Beason et al. chose arbitrary threshold value ranges based on previously reported in the literature for glenoid (25%) and humeral head (<20%, 20–40%, >40%).^55^ In agreement with previous studies, Ozaki et al. state that many reports suggest a large HSL to be one of the most important risk factors for postoperative recurrence after arthroscopic Bankart repair.^22^ Critical sizes of these lesions have been reported as depths of more than 16% of the humeral head diameter, area more than 25% of the articular surface of the humeral head, and volume greater than 250 mm.^31,60–62^ Shijith et al. determined that CT is an effective modality for assessing the amount of bone loss on the glenoid side or head of the humerus, with glenoid width bone loss of more than 9.8% or Hill-Sachs defect of more than 14.8 mm being the critical defects after which the frequency of dislocations increases (Table 7).^39^

**Table 7.** Detecting the presence of Hill-Sachs lesions compared with various modalities.

Article	Modality	Reference test	Quantification method	Sensitivity, %	Specificity, %	Accuracy, %	PPV, %	NPV, %	Intrarater agreement	Interrater agreement
Beason A.M. (2019)	Plain radiographs and MRI	NR	NR	NR	NR	NR	NR	NR	Radiographs = k of 0.48 MRI = 0.52	Radiographs = 0.35 MRI = 0.33
Chalmers P.N. (2020)	MRI vs CT	NR	Linear-based and area-based methods	NR	NR	NR	NR	NR	% Agreement CT on track = 33.0% % agreement MRI on track = 40.5% % Agreement combined = 83%	Combined bone loss MRI ICC = 0.57, 95% CI of 79% CT ICC = 0.62 95% CI of 83%
Stillwater L. (2017)	3D-MR vs 3D-CT	NR	Maximal humeral head height (A) Residual humeral head width (B) Percentage humeral head bone loss = [(A–B/A)*100]	NR	NR	NR	NR	NR	NR	NR
Pavic R. (2013)	Ultrasound (US) MRI MRA	Arthroscopy	NR	NR	NR	NR	NR	NR	NR	NR
Oh J.H. (2010)	CTA and MRA	Arthroscopy	NR	93.0	90.0	90.0	67.0	98.0	NR	NR
Breighner RE (2018	CT and MRI (ZTE)	NR	Measured in radial and tangential directions depth and width, respectively	NR	NR	NR	NR	NR	NR	K = 0.76 K = 0.7 (ZTE)
Farin PU (1996)	US and CTA	Arthroscopy	Normal base area inspected for diagnosis	91.0	95.0	94.0	NR	NR	NR	NR
Simão et al (2012)	US and MRA	Arthroscopy	NR	MRA = 100% US = 44% to 90%	MRA = 60% to 100% US = 60%	MRA = 100% US = 50% to 74%	MRA = 100% US = 67% to 82%	MRA = 100% US = 38% to 75%	K = 0.25 and 0.65	US: K = 0.4 (Fair) MRA: K = 0.64 (Good)
Sgroi et al. (2021)	CT and MRI	Arthroscopy	Width and depth, Franceschi grading, Calandra classification, Richards grading, Hall grading, Rowe grading, Flatow percentage, Glenoid track assessment	NR	NR	NR	NR	NR	NR	NR
Riebe et al. (2021)	Radiographs	MRI	Height, width, and depth	72.9	NR	NR	NR	NR	NR	CT: Franceschi (ICC = 0.359) and Calandra (ICC = 0.361) classifcations (poor) All other measurements showed good (glenoid track, α = 0.632) or excellent reliabilities MRI: Franceschi (ICC = 0.120) and Calandra (ICC = 0.154) (poor), all others showed fair (glenoid track, α = 0.413) or excellent reliabilities
Yu et al. (2020)	Radiographs	MRI	Broken circle method	71.5	95.2	77.4	97.9	54.1	NR	NR

NR: not reported.

## Discussion

In the current review, there is significant variability in imaging modality and measurement techniques, with MRI and depth being the most prevalent, respectively. The current literature on the assessment of HSL demonstrates a wide range of measurement techniques and imaging modalities with support for MRI and MRA. However, results should be taken with caution due to the small number of included studies with each modality, the variability in study designs, and the lack of high-quality and comparative studies. In addition, the variety of measurement techniques corroborate the lack of standardization and agreement regarding the best modality and measurement method, suggesting that at this point there is no clear superiority of one imaging modality or measurement technique above the other.

MRA showed the highest sensitivity and specificity values amongst the different imaging modalities, but with values that range considerably from 69% to 100%.^24,26,32,34,46,49^ Accuracy (81–100%), and intra-rater and inter-rater agreement was highest amongst MRA compared to all other modalities. MRA is reliable in diagnosing various shoulder pathologies such as intra-articular cartilage and ligaments injuries, labral tears, and rotator cuff disease amongst others.^63^ MRA can also be effective in measuring HSLs in adolescent patients and can help address bony complications of HSLs to accurately assess the lesion.^59^ Another consideration is the viewing angle of the MRI. For example, the abduction-external rotation view and the apprehension test position are both recommended as useful techniques for detection of anterior shoulder instability, with the latter being possibly more beneficial in HSL examination when using indirect arthrography.^35^ Despite its numerous benefits, MRA imaging has its drawbacks, most notably it is an expensive imaging tool. In addition, metal in the vicinity of the lesion can interfere with the true signal.^30^ Furthermore, the reproducibility and accuracy of MRA assessments is moderate.^32^

Many studies have shown success with measuring HSLs with other modalities, most notably standardized CT protocols. In fact, most clinicians use CT imaging as part of their preoperative assessment process when dealing with patients with shoulder instability. However, given the variability and inconclusive results among CT studies in this review, it is difficult to conclude its reliability despite its use by clinicians. Another consideration is the potential radiation exposure patients may experience during CT imaging.^58,59,64^ Fortunately, newer CT protocols have shown a reduction in radiation exposure by developing low dose scans protocols, therefore decreasing this concern.^65,66^ There exists a need to conduct analyses to directly compare imaging modalities not only regarding accurate measuring of HSLs, but also their safety profile and potential exposure risks to patients.

In 3D-CT HSLs measurement methodologies, analysis of a two-dimensional image of a three-dimensional object leaves many discrepancies. This often leads to misinterpretation in raters from image imperfections or measurement errors.^8^ However, 3D imaging adds the benefit of modeling which can show the nature of the defect alongside the location for considerations of operative repair.^21^ MR imaging more accurately quantifies Hill-Sachs interval as the rotator cuff insertion is more clearly visible than with CT scans and allows for evaluation of soft-tissue injuries accompanying primary anterior shoulder instability.^58^

Unfortunately, accurate measurement of HSLs volume is often difficult. The wide variety of measurement methods reflect the lack of agreement in this area, although imaging findings do not always reflect what is observed arthroscopically.^59,67^ Recently, arthroscopic evaluation has been questioned due to poor accuracy and reliability when compared to CT scans with the potential of overestimating bone defects in patients with glenoid bone loss.^68^ Thus, even when the most commonly used reference or gold standard test in this scoping review was arthroscopy in around 85% of the studies, there are concerns about its precision.

Preoperative planning in glenohumeral instability plays a pivotal role in determining appropriate treatment plans for patients, therefore analyzing imaging modalities and measurements of glenoid and humeral bone loss is essential for the treatment decision-making process. Among the 45 studies, CT-based tests and magnetic resonance-based tests were the most prevalent imaging modalities used as index tests. Currently there is no consensus on an accepted threshold value for HSL that will lead to a certain surgical treatment as its importance relies more in a bipolar defect concept. This creates numerous complications for quantifying bone loss, predicting engagement prior to surgery, and deciding the best treatment for anterior glenohumeral instability patients.^16^ In contrast, most of the attention has been given to quantifying glenoid bone defects. The threshold values of glenoid bone loss above which arthroscopic Bankart repairs may fail have been widely accepted as ≥25% glenoid width loss, equivalent to ≥19 % of the glenoid length and ≥20 % of the surface area created by a best-fit circle on the inferior surface of the glenoid.^16,69^ However, a better understanding of shoulder instability as a bipolar problem reflected in the glenoid track concept and its potential treatment implication warrant a more precise quantification of the HSLs to offer patients the best treatment alternative.

An analysis of the quantification methods for HSLs identified in the included studies shows that measurement of the depth of the lesion is most prevalent. HSL depth measurements have been shown in addition to other quantification methods such as length and width measurements. Given the limited quantitative data and variability in modalities used among all different techniques, difficulties arise in identifying a “gold standard” for quantifying HSLs. To address the discrepancies between preoperative and intraoperative measurements of HSLs, a precise method for quantification of HSL needs to be established amongst clinicians and radiologists. Although our understanding of glenohumeral pathologies has grown exponentially, there remains a lack of consistency and agreement in the evaluation of this injury. For current surgeons, it is equally important that each technique's benefits and drawbacks are extensively studied and considered for each unique patient presentation to achieve the most accurate and best diagnosis of the HSL to dictate intervention planning. On the other hand, there is a need to establish the role of imaging modalities to optimize the decision-making process while reducing the economic burden of the healthcare system when using these resources.

## Limitations

This review consists of limitations. Firstly, a meta-analysis was not performed as there was high statistical and methodological heterogeneity among the studies and thus, results are summarized descriptively. Furthermore, although multiple imaging modalities and measurement techniques were investigated, there was a lack of a good quality and quantity of evidence available for each. Thus, our ability to comprehensively comment on a “gold-standard” and provide meaningful recommendations is limited.

High-quality comparative studies with large sample sizes should be conducted in the future to determine an optimal imaging modality and to identify the best and more effective measurement technique. Therefore, future studies should standardize assessments of accuracy and reliability for imaging modalities and measurement techniques in quantifying the HSL. Future studies should also assess how treatment decisions can change based on the use of MRI with or without MRA. Lastly, an economic/cost-benefit analysis of imaging modalities should be conducted to help guide clinicians and radiologists on what is the best modality to measure HSLs.

## Conclusion

MRA and MRI are reliable imaging modalities with good test diagnostic properties for assessment of HSLs. There is a wide variety of measurement techniques and imaging modalities for HSL assessment, however a lack of comparative studies exists. Thus, it is not possible to comment on the superiority of one technique over another. Future studies should directly compare the accuracy and reliability of imaging modalities and measurement while also conducting cost-benefit analyses.

## Supplemental Material

sj-docx-1-sel-10.1177_17585732221123313 - Supplemental material for Variability in quantifying the Hill-Sachs lesion: A scoping review [Author-notes fn1-17585732221123313] Click here for additional data file. Supplemental material, sj-docx-1-sel-10.1177_17585732221123313 for Variability in quantifying the Hill-Sachs lesion: A scoping review [Author-notes fn1-17585732221123313] by Shahrukh Khan, Ajaykumar Shanmugaraj, Haseeb Faisal, Carlos Prada, Sohaib Munir, Timothy Leroux and Moin Khan in Shoulder & Elbow

## List of Abbreviations

HSL: Hill-Sachs lesion
MRA: magnetic resonance arthrography
MRI: magnetic resonance imaging
3D-CT: 3D-computed tomography
CT: computed tomography
CTA: computerized arthrotomography
PRISMA: Preferred Reporting Items for Systematic Reviews and Meta-analyses
R-AMSTAR: Revised Assessment of Multiple Systematic Reviews
CI: confidence intervals
ICC: intraclass correlation coefficient
κ: kappa
JBJS: Journal of Bone & Joint Surgery
MINORS: methodological index for nonrandomized studies
PPV: positive predictive value
NPV: negative predictive value
P/R: notch defect/radius
D: depth of Hill-Sachs lesion
R: humeral head radius
MDC: minimal detectable change.
