# Supplementary material for: Variability in quantifying the Hill-Sachs lesion: A scoping review
Source: Shoulder Elbow. 2022 Sep 13;15(5):465–83. doi: 10.1177/17585732221123313 (PMC10557928; doi:10.1177/17585732221123313)
Supplement: sj-docx-1-sel-10.1177_17585732221123313 - Supplemental material for Variability in quantifying the Hill-Sachs lesion: A scoping review [file sj-docx-1-sel-10.1177_17585732221123313.docx]

Appendix Table 1. Search strategy

| **EMBASE:** 2527 studies | **MEDLINE:** 1281 studies | **PUBMED:** 192 studies | **COCHRANE**: 250 studies |
| --- | --- | --- | --- |
| **Strategy:**  1. exp Shoulder/ or shoulder.mp  2. glenohumeral.mp.  3. shoulder joint.mp  4. glenoid cavity.mp or exp Glenoid Cavity/  5. humeral head.mp. or exp Humeral Head/  6. 1 or 2 or 3 or 4 or 5  7. shoulder dislocation.mp. or exp Shoulder Dislocation/  8. hill-sachs bone loss.mp.  9. hill-sachs.mp.  10. bone loss.mp.  11. humeral bone loss.mp.  12. bankart lesion.mp. or exp Bankart Lesions/  13. bipolar bone loss.mp.  14. humeral lesion.mp.  15. 7 or 8 or 9 or 10 or 11 or 13 or 14  16. measurement*.mp.  17. xray.mp. or exp X-Rays/  18. exp Tomography, X-Ray Computed/ or CT.mp.  19. 3D ct reconstruction.mp.  20. exp Magnetic Resonance Imaging/ or MRI.mp.  21. 16 or 17 or 18 or 19 or 20  22. 6 and 15 and 21  23. limit 22 to (English language and humans) | **Strategy:**  1. exp Shoulder/ or shoulder.mp  2. glenohumeral.mp.  3. shoulder joint.mp  4. glenoid cavity.mp or exp Glenoid Cavity/  5. humeral head.mp. or exp Humeral Head/  6. 1 or 2 or 3 or 4 or 5  7. shoulder dislocation.mp. or exp Shoulder Dislocation/  8. hill-sachs bone loss.mp.  9. hill-sachs.mp.  10. bone loss.mp.  11. humeral bone loss.mp.  12. bankart lesion.mp. or exp Bankart Lesions/  13. bipolar bone loss.mp.  14. humeral lesion.mp.  15. 7 or 8 or 9 or 10 or 11 or 13 or 14  16. measurement*.mp.  17. xray.mp. or exp X-Rays/  18. exp Tomography, X-Ray Computed/ or CT.mp.  19. 3D ct reconstruction.mp.  20. exp Magnetic Resonance Imaging/ or MRI.mp.  21. 16 or 17 or 18 or 19 or 20  22. 6 and 15 and 21  23. limit 22 to (English language and humans) | **Strategy:**  (((((((shoulder) OR (glenohumeral)) OR (shoulder joint)) OR (glenoid cavity)) OR (humeral head)) AND (((((((((shoulder dislocation) OR (hill-sachs bone loss)) OR (hill-sachs)) OR (bone loss)) OR (humeral bone loss)) OR (bankart lesion)) OR (bipolar bone loss)) OR (humeral sided lesion)) OR (3d ct reconstruction))) AND (((((tomography) OR (CT)) OR (MRI)) OR (xray)) OR (measurement)) AND (review[Filter])) AND ((2020/09/30:3000/12/31[Date - Publication]) AND (review[Filter])) | **Strategy:**  (((((((shoulder) OR (glenohumeral)) OR (shoulder joint)) OR (glenoid cavity)) OR (humeral head)) AND (((((((((shoulder dislocation) OR (hill-sachs bone loss)) OR (hill-sachs)) OR (bone loss)) OR (humeral bone loss)) OR (bankart lesion)) OR (bipolar bone loss)) OR (humeral sided lesion)) OR (3d ct reconstruction))) AND (((((tomography) OR (CT)) OR (MRI)) OR (xray)) OR (measurement)) |
